# Landscape Fire Smoke as a Cause of Death: Burning Vegetation Estimated to Kill Hundreds of Thousands Worldwide

**DOI:** 10.1289/ehp.120-a204b

**Published:** 2012-05-01

**Authors:** Bob Weinhold

**Affiliations:** Bob Weinhold, MA, has covered environmental health issues for numerous outlets since 1996. He is a member of the Society of Environmental Journalists.

The smoke from burning forests, grasslands, agricultural areas, and peatlands contains hundreds of chemicals, and the adverse health effects of exposures to such smoke are becoming better documented. Now a team of Australian, Canadian, and U.S. researchers has calculated the first global estimate of premature deaths from all causes linked with this smoke [*EHP* 120(5):695–701; Johnston et al.].

Satellite and other data provided information on the areas burned each year over the period 1997–2006. The researchers first estimated daily and annual exposures to fine particulate matter (PM_^2.5^_), which they used as a surrogate for the many toxic substances in landscape fire smoke, distinguishing between sporadic and chronic smoke exposure. Then they used data on the health effects of smoke-related PM_^2.5^_ to estimate how many deaths could be attributed to landscape fire smoke.

The estimated annual exposure to PM_2.5_ from landscape fire smoke ranged from 0 to 45 µg/m^3^. The region with the highest population-weighted annual average was sub-Saharan Africa, at 12.2 µg/m^3^.

Over the 10-year period, the researchers estimate landscape fire smoke killed an average 339,000 people per year, with a high percentage of deaths occurring in low-income areas. By comparison, urban air pollution kills an estimated 800,000 people per year worldwide, and indoor burning of solid fuels kills an estimated 1.6 million. Strong El Niño conditions appeared to contribute to more deaths, with an estimated 532,000 deaths during the 1997–1998 El Niño cycle (compared with 262,000 during the 1999–2000 La Niña cycle).

About 46% of the premature deaths were estimated to occur in sub-Saharan Africa, and 32% were estimated to occur in Southeast Asia, each of which is home to large numbers of intentional burns for agriculture, deforestation, and other purposes. Other high-smoke areas included southeastern Russia and certain parts of Central and South America.

The study provides more insights into adverse health impacts than were previously available, but the researchers acknowledge their methods have many limitations, including uncertainties in the models used, limited data on health effects caused by landscape fire smoke, incomplete data on baseline death rates in some areas of the world, and an inability to account for factors known to alter the composition of smoke. However, the authors say there likely is enough information now available to suggest that reducing intentional fires could save many lives.

